# Real-Time Evaluation of the Mechanical Performance and Residual Life of a Notching Mold Using Embedded PVDF Sensors and SVM Criteria

**DOI:** 10.3390/s19235123

**Published:** 2019-11-22

**Authors:** Ching-Yuan Chang, Tsung-Han Huang, Tzu-Chun Chung

**Affiliations:** Department of Mechanical Engineering, National Taipei University of Technology, Taipei 10608, Taiwan; e11961933@gmail.com (T.-H.H.); as0910926022@gmail.com (T.-C.C.)

**Keywords:** preventative maintenance, support vector machine, cyber-physical system, piezoelectric films, intelligent management

## Abstract

The geometric tolerance of notching machines used in the fabrication of components for induction motor stators and rotators is less than 50 *µm*. The blunt edges of worn molds can cause the edge of the sheet metal to form a burr, which can seriously impede assembly and reduce the efficiency of the resulting motor. The overuse of molds without sufficient maintenance leads to wasted sheet material, whereas excessive maintenance shortens the life of the punch/die plate. Diagnosing the mechanical performance of die molds requires extensive experience and fine-grained sensor data. In this study, we embedded polyvinylidene fluoride (PVDF) films within the mechanical mold of a notching machine to obtain direct measurements of the reaction forces imposed by the punch. We also developed an automated diagnosis program based on a support vector machine (SVM) to characterize the performance of the mechanical mold. The proposed cyber-physical system (CPS) facilitated the real-time monitoring of machinery for preventative maintenance as well as the implementation of early warning alarms. The cloud server used to gather mold-related data also generated data logs for managers. The hyperplane of the CPS-PVDF was calibrated using a variety of parameters pertaining to the edge characteristics of punches. Stereo-microscopy analysis of the punched workpiece verified that the accuracy of the fault classification was 97.6%.

## 1. Introduction

In the fabrication of induction motors, the mechanical tolerance for component variation is very low (50 *µm*). Notched workpieces with jagged edges (burrs) can seriously undermine the assembly of the stator and rotor [1], which can compromise the efficiency of the resulting motors [2,3]. The accumulation of inaccuracies can induce backlash between sheets and produce internal strain within the stator or rotor [4,5]. The quality of notched workpieces depends on the mechanical performance of the punch/die molds, which are life-limited, i.e., the edges become blunt due to wear damage and fatigue failure. The cutting edge of sheet metal is generally analyzed via sampling after a work cycle of 10,000 ± 5% strokes to assess the mechanical performance of the die mold. Mechanical molds generally require maintenance after 500,000 ± 5% strokes, wherein high-precision grinding machines are used to sharpen the punch and to flatten the die mold. Most mechanical molds are retired after 30 ± 2 off-line maintenance sessions, due to the difficultly of correcting geometric inaccuracies. The life of the mechanical mold depends on the number of strokes (*N*) as well as the cutting shear force (*S*), which is a product of the shear strength of the sheet metal (*G*), the thickness of the steel workpiece (*T*), and the geometry of the cutting edge (*L*). The speed of the stroke (*V*) and the geometry of the punch (*P*) can also affect the life of a mechanical mold. Sampling inspections and scheduled maintenance require downtime, particularly for the estimation of the mechanical performance of the die. Researchers have used mathematical models, numerical simulations, and experimental procedures to investigate the mechanical deformation of sheet metal [6,7]. The long-term monitoring of mechanical molds involves a variety of sensors [8,9,10] to maintain a record of the cycle time, deformation, acceleration, and temperature. Many modern factories must be able to turn out a large variety of products in limited production runs with a heavy reliance on automation and smart technology, i.e., cyber-physical systems (CPSs) [11]. The accurate assessment of mechanical molds requires direct measurement of contact force with the assistance of machine learning [12,13,14]. Polyvinylidene fluoride (PVDF) film is a polymer that exhibits piezoelectric behavior after polarization [15,16]. The high β-phase and piezoelectric performance of PVDF films enables the conversion of mechanical deformations into electrical signals. These materials have been used in energy harvesters, smart sensors, shock detectors, and structural health monitoring (SHM) [17,18,19]. Conventionally, PVDF films measure out-of-plane deformation (i.e., in the thickness direction), which is optimal for pressure sensors [20,21]. Commercial PVDF films packaged with an electrode provide a convenient connection point for industrial applications; however, wire terminal connectors impose mass effects at the sensing point. Furthermore, the thickness of metal connectors generally exceeds the space available for installation in mechanical molds. In the current research, we reshaped raw PVDF films (1-1004346-0, TE connectivity, Schaffhausen, Switzerland) into rectangular devices (10 mm × 5 mm) and then used 3D printing for the attachment of wires to the enclosed PVDF sensors. Polymer layers extruded from the 3D printer covered the film and contributed to a waterproof PVDF sensor. The customized PVDF sensor was highly robust against the vibrations and other hazards typical of production lines. A charge amplifier (TE connectivity) and signal acquisition module (NI 9234, National Instrument, Austin, Texas, USA) were used to obtain dynamic responses from a notching machine. 

## 2. Experimental Setup and CPS-PVDF System

**Figure 1:**
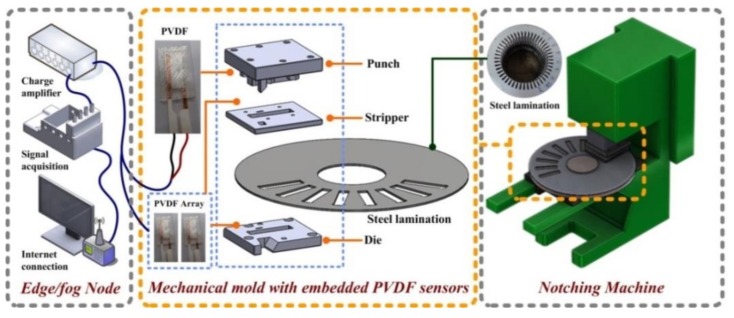
Schematic illustration of proposed cyber-physical systems- polyvinylidene fluoride (CPS-PVDF) online monitoring system: PVDF mechanical films with embedded sensors provided contact force measurements from notching machine as an indication of tool wear.

Figure 1 presents a schematic illustration of the CPS-PVDF system and online preventative maintenance program. The structure of the support vector machine (SVM) followed that of CPSs and included (1) a sensing layer with several PVDF sensors, (2) a cyber layer with a local area network (LAN) and a wide area network (WAN), and (3) an application layer with a health monitoring system. The CPS-PVDF system provides self-contained, modular, distributed, dynamic applications for working stations. We connected the CPS-PVDF system with the internet and composed a program for monitoring the overall effectiveness of the notching machine. The web service showed the vital performance of the working station, such as the residual life of the mechanical molds, the quality of the notched specimen, and the productivity of the continuous production. The big data based on precision measurement provided substantial evidence for AI prediction since the correct features obtained from the experimental results can reduce the number of the neuronal nodes and lower the computational load. 

**Figure 2:**
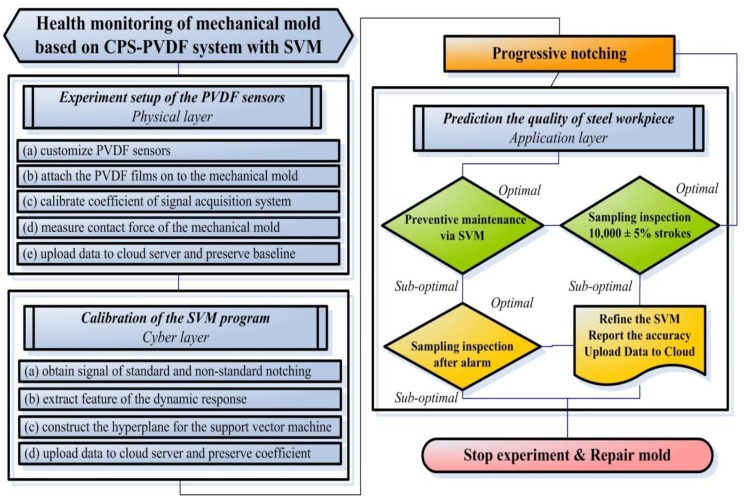
Flow chart of proposed CPS-PVDF system with SVM: customized PVDF sensors obtained direct measurements of contact force and sent dynamic signals with high SNR for interpretation via SVM and predictions pertaining to quality were verified via 3D scanning and surface profiles.

Figure 2 presents a flow chart of the proposed health monitoring system for mechanical molds used in the fabrication of thin steel workpieces. The embedded PVDF provided a high signal-to-noise ratio (SNR) for edge/fog computation, which extracts features in the time domain as well as the frequency domain. Most industrial environments are limited in terms of internet bandwidth; therefore, we put together a system that allowed a notebook computer to upload only the extracted features, rather than all of the raw data pertaining to contact force. The CPS-PVDF system included the following subroutines: (1) PVDF sensor setup corresponding to the physical layer, (2) calibration of the SVM program corresponding to the cyber layer, and (3) prediction of steel workpiece quality corresponding to the application layer. The size and shape of the PVDF sensors could be customized to minimize the time required for installation. Furthermore, there was no need to redesign mechanical molds for the attachment of sensors; the sensors can be attached to a wide range of manufacturing machines. We used the PVDF sensors to retrieve contact force data from notching machines operating with tools that were in optimal condition (sharp edges) and those that were in sub-optimal condition (blunted and/or damaged edges). The notebook computer constructed a hyperplane for the SVM program using dynamic signals related to the two operating conditions. A cloud server recorded the parameters of the extracted features for use in drawing up production history reports. The diagnosis of the mechanical mold was an implicit solution, comprising gross results pertaining to the performance of the mechanical mold, the material properties of the sheet metal, and the driving force of the punching machine. The results were applicable to the preventative maintenance of mechanical molds and in making predictions related to the quality of steel workpieces. The features used in the SVM program were as follows: (1) the average value of 42 peak-to-peak waveforms; (2) the average value of the full width at half maximum (FWHM) using 42 waveforms; (3) the first peak in the frequency spectrum; (4) the third peak in the frequency spectrum; (5) the ZNCC coefficient based on the measured signal and the pre-defined signal obtained under optimal conditions; and (6) the ZNCC coefficient of the measured signal and the pre-defined signal obtained under sub-optimal conditions.

**Figure 3:**
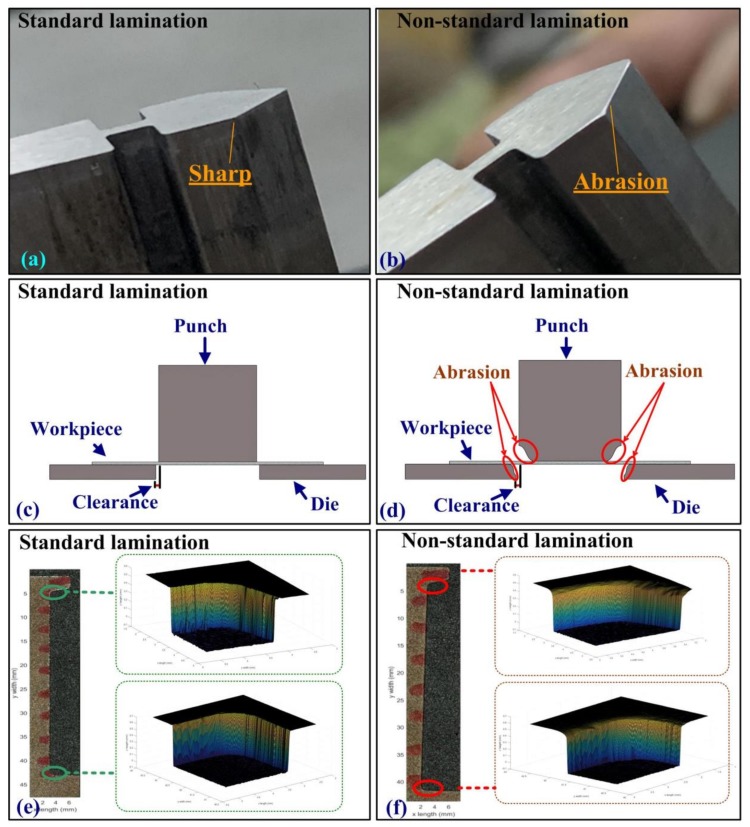
Punch in (**a**) optimal condition showing sharp edges and (**b**) sub-optimal condition showing blunt edges. Schematic illustration showing fabrication of steel workpieces using cutting edges in (**c**) optimal condition and (**d**) sub-optimal condition. Surface profile of steel workpiece fabricated using cutting tools in (**e**) optimal condition and (**f**) sub-optimal condition.

Figure 3a,b, respectively, illustrates punches in optimal and sub-optimal conditions. The two images are of the same punch before and after being subjected to edge abrasion through repeated use. Figure 3c,d presents schematic illustrations, respectively ,showing the process of punching steel workpieces using cutting edges that are in optimal and sub-optimal conditions. The thickness of the piezoelectric films was only 600 μm, which made it possible to create PVDF sensors that were flexible, compressible, and waterproof. Figure 3e,f, respectively, illustrates the surface profiles of steel components fabricated using cutting tools in optimal and sub-optimal conditions. These images were obtained using a stereo microscope (VR3100, Keyence, Osaka, Japan) to provide full-field images of the cutting edges. In most industries, the mechanical tolerance for steel workpiece components is 50 *µm*.

**Figure 4:**
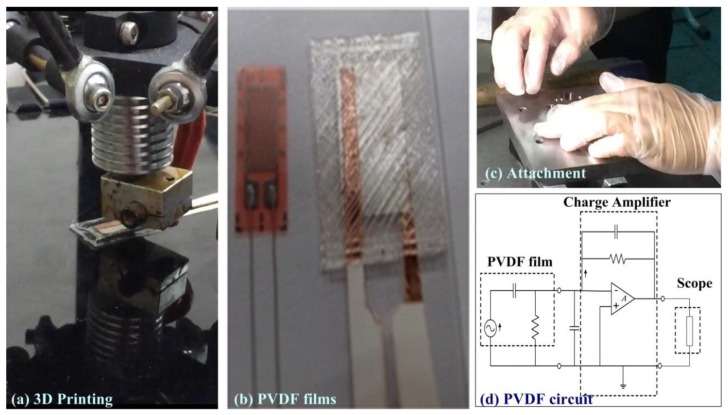
Customized PVDF film: (**a**) packaging of PVDF film sensor using a 3D printing machine; (**b**) comparison of commercial strain gauge and proposed PVDF sensor; (**c**) installation of PVDF film in mechanical mold; and (**d**) circuit used in charge amplifier.

As shown in Figure 4a, a commercial 3D printing (3DP) machine was used to cover the PVDF films with ethylene vinyl acetate (EVA). The 3DP machine was controlled using G-code written in-house. The 3DP machine, when making the cover layer, can customize the material, the thickness and the density for the bottom side and top side, as well as meet different specifications of mechanical molds. The printing temperature was maintained at 210 °C, and 6.2 s were required to print each EVA layer. As shown in Figure 4b, the customized PVDF films used in this study were 10 mm × 5 mm, which matches that of commercial strain gauges. The size of the customized PVDF was small and was close to the size of strain gauges. We then embedded the small PVDF films into the mechanical mold for retrieving the in situ signal of contact force. The thickness of the raw material used in the PVDF films was 40 *µm*. The PVDF film was used to measure out-of-plane deformation and the strain gauge was used to measure in-plane deformation. The contact force created in the notching machine was in the thickness direction of the PVDF films. We used copper foil tape as electrodes for the piezoelectric sensors to ensure a flat surface. Figure 4c illustrates the attachment of PVDF sensors to the mechanical mold. Figure 4d illustrates the circuit in which a capacitor was used to emulate the electrical properties of the PVDF film. The signal acquisition module converted analog signals into digital waveforms at a sampling rate of 10,000 *Hz*. The contact force of the mechanical mold was proportional to the measured voltage, and the configuration of the charge amplifier and the signal acquisition module was the same in all experiments. These devices also provided a high signal-to-noise ratio for fault classification. $P_{j}\left(t+i\Delta t\right)$ denotes the measured PVDF signal, where *i* is the punch cycle, *j* is the number of PVDF sensors, *t* is the time variable, and *Δt* is the cycling time required for the completion of a single workpiece.

The CPS-PVDF system provides a web service for smart devices. Software applications written in different programming languages and running on different platforms can use web services to connect industrial machines under the same LAN. The inter-process communication from device to device facilitates data exchange via the local networks. One factory contains serval stations which included multiple instruments. The proposed CPS-PVDF system promises interoperability (e.g., between Java and Python, or Windows and Linux applications) due to the customized physical layer, cyber layer, and application layer. 

**Figure 5:**
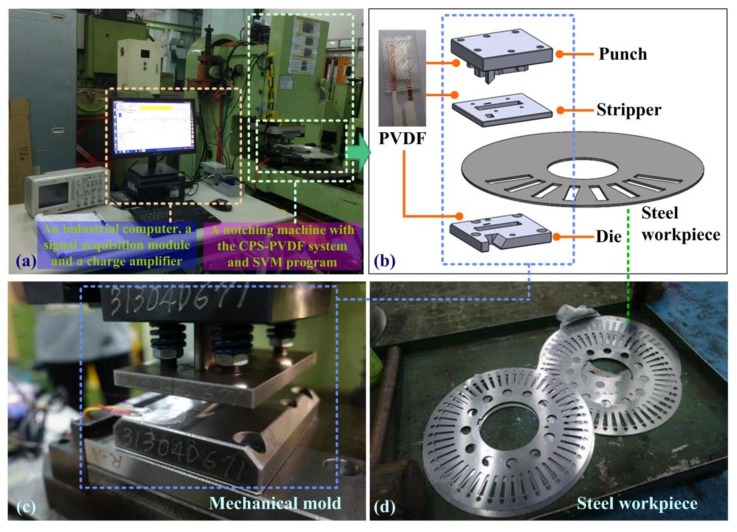
Experimental setup: (**a**) notching machine equipped with proposed PVDF film sensor; (**b**) practical setup of mechanical mold; (**c**) mechanical mold with embedded PVDF sensors and (**d**) completed workpiece.

Figure 5 presents the experiment setup of the proposed CPS-PVDF system with SVM, which included a mechanical mold, PVDF films, a signal acquisition module with charge amplifier, and a notebook computer. The thickness of the PVDF sensors was thin enough to allow installation within a mechanical mold. The measured voltage was linear proportion to the contact force. In this study, customized PVDF sensors were embedded within the mechanical mold. Figure 5a illustrates the implementation of the proposed system for long-term observation, i.e., greater than 15,000 strokes. The CPS-PVDF system was implemented in an electric motor factory to facilitate preventative maintenance of a mechanical mold. Figure 5b presents the mechanical mold, which includes a punch, a stripper, and a die plate. An automatic feed system moved the metal into notching position between the stripper and die. Figure 5c presents the punched steel workpieces, which have been inspected after regular period of notching time.

The notching machine provided 290 strokes per minutes (SPM), and the sampling rate of the PVDF film was 10,000 points per s. The embedded PVDF sensors could sustain over 10,000 strokes and demonstrated the potential of industrial measurement. This work upgraded the mechanical mold from a sensorless device to an Internet of things (IoT) component. The edge computer evaluated the performance of the die mold instead of manual diagnosis. The CPS-PVDF system cooperated with different language environments: MATLAB collected spectrum data from the PVDF sensor; LabVIEW recorded waveform from the PVDF films; and Python encrypted, compressed, and uploaded the experimental results. The customized format of the experiment data provides high flexibility for various sensors and the database on the cloud server is expandable for streaming data. The Internet can create active server pages (ASP) and would allow the calculation of the gross productivity of a factory located in a suburban region. This would allow general users to subscribe to standard information with limited conditions, and authorized analysists all around the world to receive rapid and complete information, even in different time zones.

## 3. Preventative Maintenance of Mechanical Molds Based on SVM Criteria

**Figure 6:**
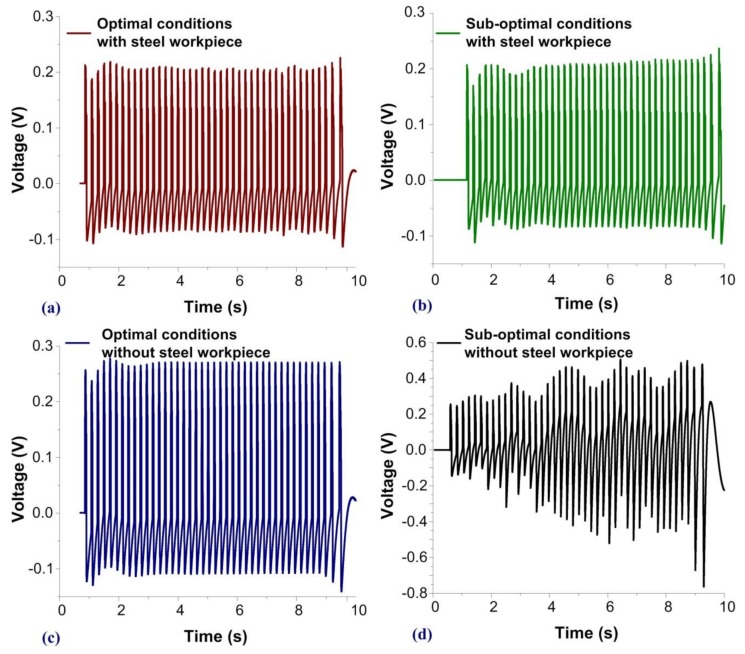
Comparison of PVDF waveforms at macroscale: signals obtained with a steel workpiece loaded into the notching machine with (**a**) sharp edges and (**b**) blunt edges; and signals obtained without a steel workpiece loaded into the notching machine with (**c**) sharp edges and (**d**) blunt edges.

Figure 6 presents contact force measurements and a macroscale comparison corresponding to four fabrication conditions. The first and second columns respectively list the results obtained using (1) a punch in optimal condition (sharp) and (2) a punch in sub-optimal condition (blunt). The first and second rows, respectively, show the results obtained (1) when a component of a steel workpiece was loaded in the machine and (2) the results obtained when no steel workpiece was loaded in the machine. The notching machine was operated at 290 strokes per minute (SPM) and each component required 42 strokes to complete fabrication. Thus, the time required for the completion of a single specimen was 10 s. The SVM program extracted features from the obtained waveforms and spectra for the construction of a hyperplane to classify the punch/die as sharp or blunt. Figure 6a presents the force measurements obtained during the notching of a steel workpiece under optimal tooling conditions (sharp) and the profile is presented in Figure 3e. Figure 6 shows the measurements obtained after the mechanical mold had completed 15,000 strokes, wherein the edge of the punch had become blunt, which resulted in entirely different waveforms. Figure 6c,d, respectively, presents the force measurements obtained during the operation of machines without any steel workpiece loaded in the machine. The contact force seen in Figure 6c clearly exceeds that seen in Figure 6d. The blunt punch head required greater force to slide through the die hole. These results demonstrate the high sensitivity and high SNR of the measurement signals obtained using the embedded PVDF films. 

**Figure 7:**
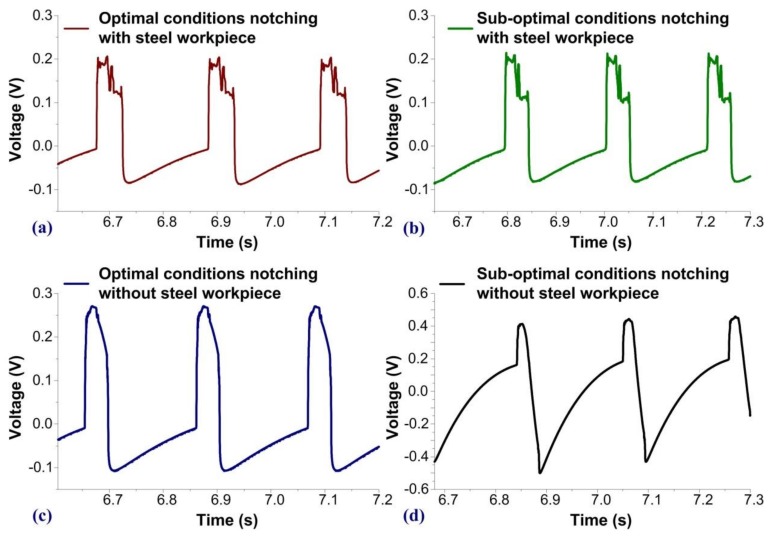
Comparison of PVDF waveforms at microscale: signals obtained with a steel workpiece loaded into the notching machine with (**a**) sharp edges and (**b**) blunt edges; and signals obtained without a steel workpiece loaded into the notching machine with (**c**) sharp edges and (**d**) blunt edges.

Figure 7 presents a comparison of force measurements at the microscale, where the time of observation was 0.6 s. The time origins in the four experiments differed slightly; however, the discrepancies were less than 0.05 s. Figure 7a,b, respectively, presents the waveforms obtained when the tools were in optimal and sub-optimal condition and a steel workpiece was loaded in the machine. Figure 7c presents the waveforms obtained under optimal conditions without a steel workpiece loaded in the machine, i.e., the characteristics of the machine operating without stress. This waveform was smoother than that shown in Figure 7a, in which the punch passing through the steel workpiece created high-frequency responses in the sensors. Figure 7d presents the waveform obtained under sub-optimal conditions without a steel workpiece loaded in the machine. In this case, the slope and the peak values of the waveform were higher than those seen in Figure 7c. 

**Figure 8:**
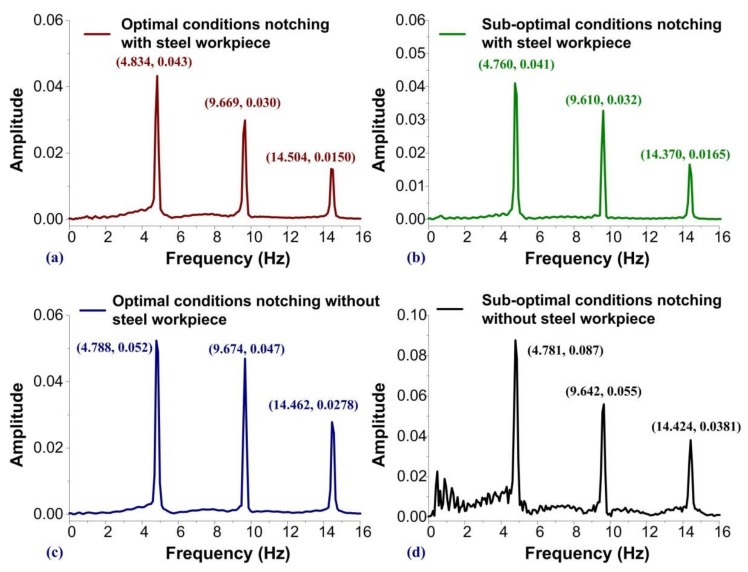
Comparison of the frequency spectra of measured PVDF signals: signals obtained with a steel workpiece loaded into the notching machine with (**a**) sharp edges and (**b**) blunt edges; and signals obtained without a steel workpiece loaded into the notching machine with (**c**) sharp edges and (**d**) blunt edge.

Figure 8 presents the frequency spectra derived from the PVDF sensors during an observation period of 10 s. The frequencies and peak values indicate the dynamic features of the contact forces under various operating conditions (at 290 SPM). Figure 8 presents the frequencies of the first peaks at 4.834, 4.760, 4.788, and 4.781 *Hz*. The drift ratio of the first three peaks obtained from the measured frequencies was small and was related to the mechanical performance of the punch/die during operations. We employed evidence-based features for fault detection as an alternative to multiple layers of invalid features, which would have required extensive training data and would have imposed a heavy computational burden. The program classified the condition of the mold in terms of sharpness or abrasion. The SVM function was designed to extract waveforms from the contact force measurements $P_{j}\left(t+i\Delta t\right)$ as well as the spectra of the dynamic responses using the fast Fourier transform (FFT). The symbol *F* denotes the operation of feature extraction:

(1) $$Q\left(i,j,k\right)=F\left\{P_{j}\left(t+i\Delta t\right)\right\},$$

where the free index *k* denotes the notable features related to contact force in the temporal domain and in the frequency spectrum as well. The sizes of *i*, *j*, and *k* are indicated by *I*, *J*, and *K*, respectively. Edge/fog computation was conducted using zero mean normalized cross-correlation (ZNCC) to compute the degree of similarity between the measured signals and optimal/sub-optimal signals. The mathematical model of ZNCC is written as follows:

(2) $$H_{j}\left(t\right)=\left(P_{j}\left(t\right)*f\left(t\right)\right)=\int \frac{\left[P_{j}\left(t\right)-{\bar{P}}_{j}\left(t\right)\right]\left[f\left(t+\tau \right)-\bar{f}\left(t\right)\right]}{{\bar{\bar{P}}}_{j}\left(t\right)\bar{\bar{f}}\left(t\right)}dt\;\mathrm{and}$$

(3) $${\tilde{H}}_{j}\left(t\right)=\left(P_{j}\left(t\right)*g\left(t\right)\right)=\int \frac{\left[P_{j}\left(t\right)-{\bar{P}}_{j}\left(t\right)\right]\left[g\left(t+\tau \right)-\bar{g}\left(t\right)\right]}{{\bar{\bar{P}}}_{j}\left(t\right)\bar{\bar{g}}\left(t\right)}dt,$$

where $P_{j}\left(t\right)$ is the clipped signal from the measurement results;$f\left(t\right)$ and $g\left(t\right)$ are the pre-defined waveforms obtained under optimal and sub-optimal conditions; $\bar{f}\left(t\right)$ and $\bar{\bar{f}}\left(t\right)$ are the mean value and the standard deviation of $f\left(t\right)$; $\bar{g}\left(t\right)$ and $\bar{\bar{g}}\left(t\right)$ are the mean value and the standard deviation of $g\left(t\right)$, respectively. The SVM program presented the extracted features in a vector form as follows:

(4) $${\vec{x}}_{i}=\left(Q\left(i,1,1\right),Q\left(i,1,2\right),\cdots Q\left(i,1,K\right),\cdots Q\left(i,J,K\right)\right).$$

The results obtained under optimal and sub-optimal conditions yielded $y_{i}=1$ and $y_{i}=-1$, respectively. The experimental results of $\left({\vec{x}}_{i},y_{i}\right)$ were used to construct a hyperplane for the support vector machine. The normal vector of the hyperplane $\vec{w}$ gives:

(5) $$\vec{w}\cdot {\vec{x}}_{i}-b,$$

where *b* is the distance between selected points and the SVM hyperplane. The SVM-PVDF can be used for the online monitoring of a variety of mechanical molds.

**Figure 9:**
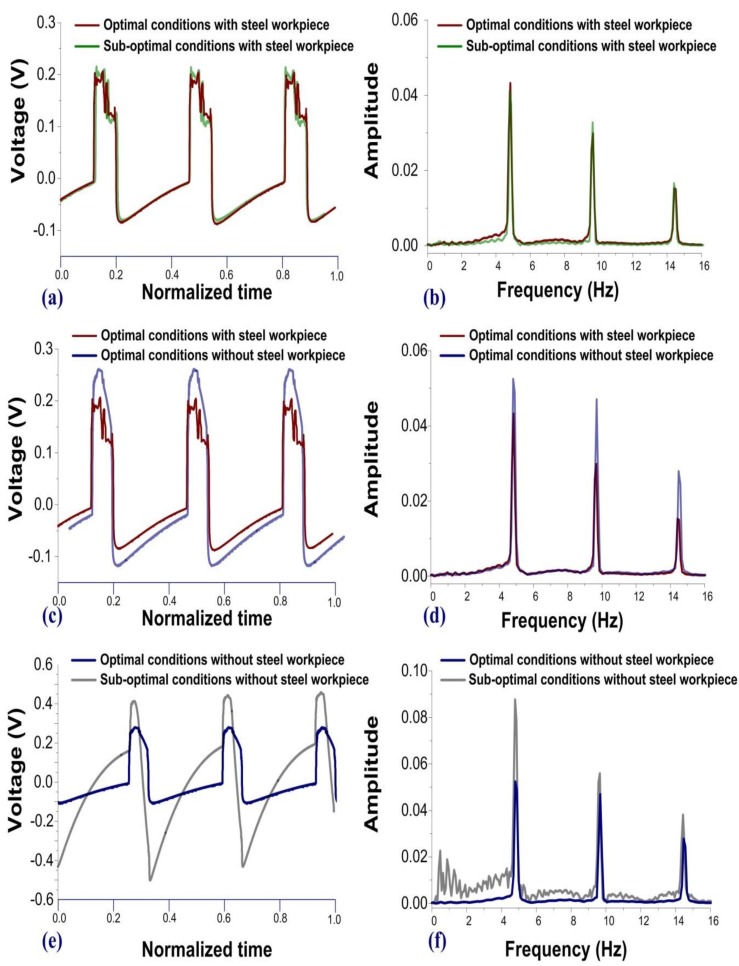
Results of comparison between (**a**,**c**,**e**) time domain waveforms and (**b**,**d**,**f**) FFT spectra: (1) a mechanical mold with blunt edges required greater cutting force than did a mold with sharp edges; (2) loading a steel workpiece induced a high-frequency component, regardless of edge condition; and (3) operating the machine without a steel workpiece provided a high SNR ratio, regardless of edge condition.

Figure 9 presents a comparison of the waveforms and FFT spectra. Figure 9a,c,e translates and stretches the time axis to give a straightforward comparison of the waveforms. Figure 9b,d,f compares the FFT spectra, which can indicate differences between waveforms. Based on experimental results in the time and frequency domains, we came to the following conclusions: (1) operating the mechanical mold with a blunt punch required a higher cutting force than with a sharp punch; (2) operations with a steel workpiece loaded in the machine induced additional high-frequency responses, regardless of whether the tools were in optimal/sub-optimal conditions; and (3) operations without a steel workpiece loaded in the machine provided a higher SNR than those signal obtained from the condition with steel workpiece. The fault diagnosis used in this study was based on the condition without steel workpieces.

**Figure 10:**
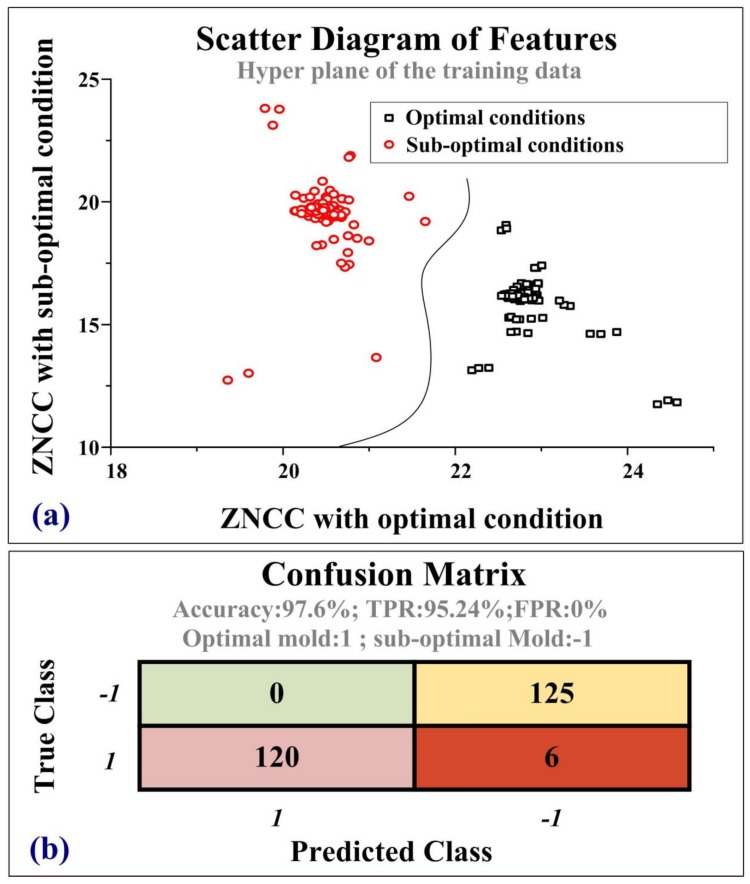
SVM program for the identification of sharp and blunt edges: (**a**) scatter plot of features in training data and (**b**) confusion matrix of measured signal; fault classification based on zero mean cross-correlation to ensure high accuracy for industrial applications.

Figure 10 presents the results computed by the SVM, wherein the black dot denotes optimal (sharp) operating conditions and red dots denote sub-optimal (blunt) operating conditions. Figure 10a presents the scatter diagram of features, where the X-axis and Y-axis denote the ZNCC obtained under optimal and sub-optimal conditions, respectively. The clear border of the hyperplane is an indication of high classification accuracy. Figure 10b presents the confusion matrix of the SVM program. This indicates that the classification accuracy was 97.6%, which is high enough for almost all industrial applications.

**Figure 11:**
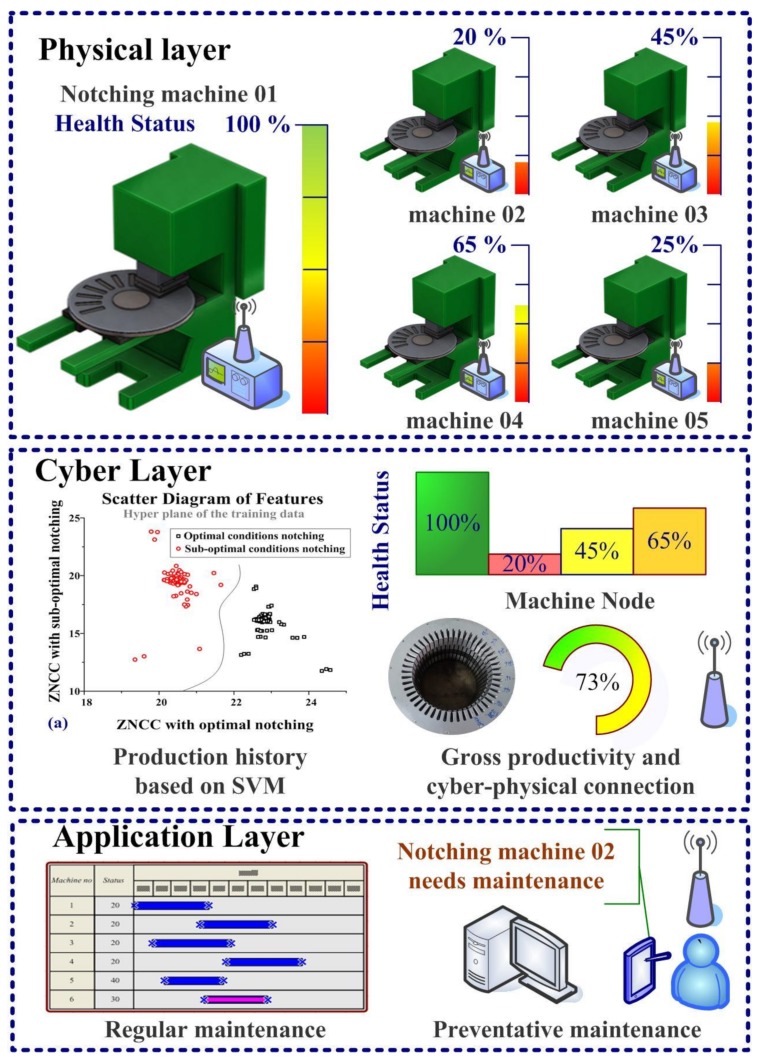
The proposed CPS-PVDF system provided visualized results of gross productivity and arranged a schedule of preventative maintenance. The cloud server (cyber layer) enabled health monitoring for multiple notching machines and sent routine messages to the administrator to facilitate management.

Figure 11 illustrates the CPS-PVDF system, which supported a range of pre-defined features for notching machines in standalone or production chain configurations. Furthermore, the SVM database was reusable and expandable. The proposed SVM-PVDF system is an example of a CPS involving the conversion of mechanical molds into smart molds. The CPS-PVDF could be used for the online monitoring of a variety of mechanical molds, where the cloud server arranges a schedule for preventative maintenance and calculates the gross productivity of the production chain. In the application layer, the CPS-PVDF system sent an optimized plane to the manager for preventative maintenance. Regular maintenance generally focuses on reshaping the cutting edges; however, the scheduling of such maintenance is largely a matter of trial-and-error based on the experience of the operator. The proposed CPS-PVDF system also sent alarm messages to the manager if the residual strokes of a specific machine are lower than a pre-defined threshold. In a conventional factory setting, the surface profiles of random samples undergo off-line evaluation to gauge the mechanical performance of the punch/die mold. The number of training and test samples was 236 and 251, respectively. After the training process, The CPS-PVDF system with SVM can evaluate the sharp/blunt edge of mechanical molds. We used a confusion matrix to describe the efficiency of the proposed SVM criteria. The row denotes the true class obtained from experimental results, and the column means the predicted class given from the SVM program, respectively. The true-positive rate (TPR) was 95.24%, and the false-positive rate (FPR) was 0%. The accuracy was (245/251) is 97.6%, which provides high efficiency for industrial applications.

## 4. Conclusions

In this study, we customized PVDF sensors and retrieved contact force measurements during the stamping of a steel workpiece. A cam mechanism in the notching machine controlled the working displacement in the cutting of the sheet metal. An SVM program extracted patterns from this raw data to differentiate between situations involving sharp cutting tools and those performed using blunt cutting tools. Steel workpieces are core components of induction motors, and the geometric tolerance of the notched workpieces dominates the gross performance of the electromechanical machines. This research proposed a CPS-PVDF system with SVM criteria to evaluate the mechanical performance of notching machines. The in situ measurement of contact force enabled the classification of the shape/blunt status of the punching molds. The contribution of the article was to provide cost-effective metrologies for the production lines. The peak-to-peak values corresponded to the physical features of the contact force. The feature used in the SVM program was based on the direct measurement of contact force and this study decrypt the physical meaning related to sharp or blunt punch for the industrial applications. The high accuracy and small differences detected in the frequency domain were subsequently used in the SVM program. Each experiment involved 42 strokes; therefore, the SVM program sliced the measured waveforms into 42 individual segments for the computation of ZNCC. This work contributes to the early detection of abrasion seen in the mechanical molds and avoids downtime costs from unexpected failures. The ultimate objective was to maintain cutting quality while optimizing maintenance schedules. Stereo microscopy verified that the accuracy of the proposed scheme exceeded 95%. The proposed system is a highly cost-effective approach to real-time inspections and the long-term monitoring of industrial machinery.
